# (Cost)-effectiveness of family meetings on indicated prevention of anxiety and depressive symptoms and disorders of primary family caregivers of patients with dementia: design of a randomized controlled trial

**DOI:** 10.1186/1471-2318-8-2

**Published:** 2008-01-21

**Authors:** Karlijn J Joling, Hein PJ van Hout, Philip Scheltens, Myrra Vernooij-Dassen, Bernard van den Berg, Judith Bosmans, Freek Gillissen, Mary Mittelman, Harm WJ van Marwijk

**Affiliations:** 1 Department of General Practice, Institute for Research in Extramural Medicine, VU University Medical Center, Amsterdam, The Netherlands; 2 Department of Neurology and Alzheimer Center, VU Medical Center, Amsterdam, The Netherlands; 3 Alzheimer Centre UMC Nijmegen, Nijmegen, The Netherlands; 4 Health Technology Assessment Unit, Institute for Research in Extramural Medicine, VU University Medical Center, Amsterdam, The Netherlands; 5 Department of Psychiatry, New York University School of Medicine, New York, USA

## Abstract

**Background:**

Dementia is a major public health problem with enormous costs to society and major consequences for both patients and their relatives. Family members of persons with dementia provide much of the care for older adults with dementia in the community. Caring for a demented relative is not easy and fraught with emotional strain, distress, and physical exhaustion. Family caregivers of dementia patients have an extremely high risk developing affective disorders such as major depression and anxiety disorder. Family meetings appear to be among the most powerful psychosocial interventions to reduce depression in caregivers.

An American landmark study reported substantial beneficial effects of a multifaceted intervention where family meetings had a central place on depression in family caregivers as well as on delay of institutionalization of patients. These effects were not replicated in other countries yet. We perform the first trial comparing only structured family meetings with significant others versus usual care among primary family caregivers of community dwelling demented patients and measure the effectiveness on both depression and anxiety in the primary caregiver, both on disorder and symptom levels.

**Methods/design:**

In this randomized controlled trial effectiveness as well as cost-effectiveness of family meetings is evaluated. The intervention group receives four family meetings with family and close friends of the primary family caregiver of a community dwelling patient with a clinical diagnosis of dementia. Dyads of patients and their primary caregiver are followed up to one year after baseline assessment. The main outcome measures are the incidence of anxiety and depressive disorders assessed with the Mini-International Neuropsychiatric Interview (MINI) and the severity of anxiety and depressive symptoms in caregivers is measured by validated self report instruments: the Centre for Epidemiologic Studies Depression Scale (CES-D) for depression and the anxiety scales of the Hospital Anxiety and Depression scales (HADS) for anxiety. The economic evaluation is performed from a societal perspective.

**Discussion:**

By evaluating the effectiveness of only structured family meetings organized in the Netherlands, this study will contribute to the existing literature about the value of psychosocial interventions for dementia caregivers.

**Trial registration:**

Dutch Trial Registry ISRCTN90163486

## Background

Dementia is a major public health problem. The estimated prevalence rate of dementia among community dwelling elderly aged 65 to 95 is 6.6% [1]. A recent paper reported that approximately 5.1 million people in the European Union live with dementia. Within the next 50 years this number will rise to approximately 11.9 million cases [2]. Because most people with dementia have a supporter or caregiver the number of family caregivers caring for a demented person will increase substantially as well.

Dementia is a syndrome with major consequences for both patients and their relatives. Caring for a demented relative is not easy and fraught with emotional strain, distress, and physical exhaustion. Previous studies showed the negative psychological, physical and social consequences associated with providing care to a relative with AD [4-6].

Family caregivers of dementia patients have an extremely high risk developing affective disorders such as major depression and anxiety disorder [3,7]. The month prevalence of depressive disorders among caregivers varied between 15–32% in three representative community samples, the 12 months incidence of depressive disorders was 48% as reported in one representative sample [7,8]. Data on anxiety disorders in dementia caregivers are scarce but suggest that 1 in 3 caregivers suffers from an anxiety disorder [9]. Depressive and anxiety symptoms are even more prevalent and affect between half to three-quarter of all caregivers [7,8,10]. The risk to develop an affective disorder persists over many years of caregiving and even after caregiving ends with the death of a care recipient [3,11].

Despite a wide range of useful services that can help patients and relief caregivers, the burden of caregiving remains very high [3]. Long term institutionalization of demented patients is extremely costly. The mental health of the family caregiver is often decisive in the timing of nursing home placement. Any intervention that can relief the burden of caregiving and (thereby) prevent mental health problems of caregivers is important. In addition, if such efforts can delay nursing home placement they are likely to be extremely cost-effective.

Despite dementia caregivers often providing intensive levels of assistance and reporting low levels of life satisfaction and high levels of overload, their use of support services is low. Perceived lack of need or lack of awareness are the main reasons for this non-use of services [12]. An empowering intervention like family meetings seems to connect very well by the demand of many caregivers to solve things on their own.

Moreover, psycho-education, an essential component of the family meetings, shows consistent effects on various outcomes like caregiver burden, depression and subjective well-being [13]. The involvement of other family members in the intervention may in all probability strengthen these positive effects.

Our hypothesis is that affective disorders of dementia caregivers are largely preventable. Systematic reviews and meta-analyses show that information and support alone is helpful but only address the psychological needs of caregivers modestly at best [13,14]. Programs that demonstrate beneficial effects on affective disorders involved both patients and their families, are more intensive and modified to caregivers needs [14,15]. As demonstrated in the landmark studies of Mittelman et al., family meetings, designed to mobilize support of naturally existing family networks, appear to be among the most powerful psychosocial interventions to reduce depression in caregivers [16-19]. However, no studies have evaluated the preventive effects on anxiety. Family counseling can maximize the positive contributions of each member to caregiving, prevent one member from carrying the entire weight of the caregiving role, improve the caregiver's understanding of how to ask for help, what kind of help is reasonable to expect from family members, and how to accept help, and reduce family conflict. In addition, these programs resulted in postponement of nursing home placement of patients [17,20].

However, the effectiveness of using only the family counseling component of this intervention has not been tested. Furthermore, effects of family meetings were not replicated in other countries yet. Therefore, inspired by the New York University Spouse-Caregiver Intervention Study, the Department of General Practice of the VU Medical Center developed an innovative intervention to support primary family caregivers of a community dwelling patient with a clinical diagnosis of dementia.

Four meetings with family and close friends will be organized and run by a trained counsellor according to a manual [21] and additional information from in depth interviews with the original counselors. The aim is to offer psycho-education, increase problem solving skills and mobilize the naturally existing social network of patient by sharing support tasks of network members. A randomized controlled trial is performed to investigate the effectiveness and cost-effectiveness of family meetings on indicated prevention of anxiety and depressive disorders and symptom levels of primary family caregivers of patients with dementia.

By evaluating the effectiveness of only structured family meetings organized in the Netherlands, this study will contribute to the existing literature about the value of psychosocial interventions for dementia caregivers. In addition, this study involves some other innovative elements. The New York University Spouse-Caregiver Intervention [22] was done with spouse caregivers of Alzheimer type dementia patients only and measured effects on depression but not on anxiety. In our study we also include other caregivers relations (e.g. child, brother/sister), but this caregiver has to live together with the patient. We expect this group to be particularly burdened and therefore vulnerable to develop a depression or anxiety disorder. Furthermore, we include also patients with non-alzheimer type dementias and measure both depression and anxiety in the caregiver.

The objective of this paper is to describe the study protocol of this RCT among primary family caregivers of dementia patients. The main research questions concern whether structured family meetings are more (cost)-effective than usual care in preventing caregivers of depression or anxiety disorders, decreasing severity of symptoms and caregiver burden, increasing caregiver's and patient's quality of life, and in increasing days until institutionalization.

## Methods/design

### Design

**Figure 1:**
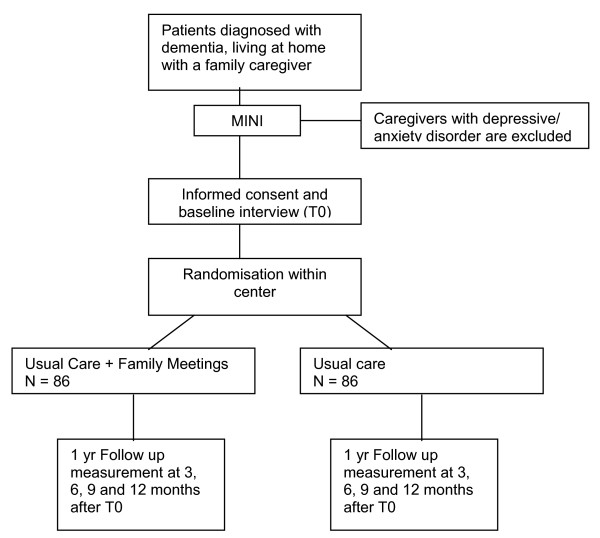
Flowchart of study design.

The design is a randomized controlled indicated prevention trial (Figure 1). After selection, and baseline assessment, dyads of patients and their primary family caregiver are randomized to one of two groups; the intervention group receives usual care plus family meetings, the control group receives usual care only. Participants are free to seek additional assistance and support elsewhere at any time throughout 12 months follow-up. Participants are only allowed to enter the study after signed informed consent. In case of mental incompetence of a patient the family caregiver will sign the consent for the patient. The Medical Ethics Committee of the VU University medical centre in Amsterdam has approved the study (ref. no 2007/83). The study is registered as clinical trial under ISRCTN90163486.

### Participants

Caregivers and patients are recruited through various memory clinics and specialized mental health care institutions. Caregivers are potentially eligible if they are a primary caregiver of a non-institutionalized relative with a clinical diagnosis of dementia and lives in the same region as the patient. In each family, at least one other family member lives in the same region of the patient and caregiver. If the dementia patient has more than one family caregiver, the primary caregiver is the one who coordinates the caring process, mostly the person who spends the most hours on caregiver tasks. We only include spouses, children (-in-law) and brothers/sisters living with the patient.

Patients and caregivers will be excluded when having severe somatic or psychiatric co-morbidity which will significantly impair cooperation to the program. Because the aim is to prevent caregivers for developing an anxiety or depressive disorder the incidence of anxiety and depressive disorders is our primary outcome. Therefore we have to exclude caregivers who already have a depressive or anxiety disorder. To detect prevalent cases caregivers are screened before baseline assessment with the MINI. They will also be excluded when either caregiver or patient participates in other intervention studies at inclusion or during the study. The in- and exclusion criteria are listed in Table 1.

**Table 1:** Inclusion and exclusion criteria

**Inclusion criteria**
▪ Family caregiver:
- takes primary responsibility for the family care of a community dwelling patient with a clinical diagnosis of dementia
- is spouse, child (-in-law) or brother or sisters of the patient.
- lives together with the patient
▪ In each family, at least one other family member or close friend lives in the same region of the patient and caregiver.
▪ Both caregiver and patient have sufficient language proficiency in Dutch for adequate participation in meetings, interviews and tests.

**Exclusion criteria**

▪ Severe somatic or psychiatric co-morbidity of either caregiver or patient, which will significantly impair cooperation to the program.
▪ Caregiver has a depression or anxiety disorder at baseline
▪ Either caregiver or patient participates in other intervention studies at inclusion or during the study which may interfere substantially with the study outcomes.
▪ The patient is scheduled to move a nursing home in short notice.

### Randomization

Randomization takes place after baseline measurement. An external person independent from our study group establishes the random order using random number tables. Blocking by center is used to ensure that comparison groups are of approximately the same size per center.

### Intervention

Primary caregivers in the intervention group will participate in four meetings with family and close friends which will be organized and run by a counselor with an advanced degree in nursing, social work, psychology or allied professions. The four family counseling sessions will follow the manual inspired by the New York University Spouse-Caregiver Intervention Study [21] and additional information from in depth interviews with the original counselors. This information clarifies and illustrates the basis principles of the intervention. Counselors have received a training prior to the study. One counselor is assigned to one caregiver to establish an ongoing relationship with someone who is familiar with the situation. Every site will pilot some meetings before the effect study will start. The aims of the family meetings are: 1) to educate families about dementia consequences and resource information for care support; 2) to mobilize naturally existing family networks of patient and primary caregiver in order to improve emotional and instrumental support; 3) to teach problem solving and techniques for troublesome patient behavior that can be used after family counseling ends. The content of the sessions is also determined by the needs of each caregiver. For example, learning techniques, for management of troublesome behavior, promoting communication between family members. All sessions are audio taped for supervision, intervision and detailed analysis. Besides, for every meeting the counselor fills in a standardized report for process evaluation.

### Usual care

The diagnostic assessments at memory clinics in the Netherlands lead to a disclosure meeting with the patient and in most cases the primary family caregiver. Patients diagnosed with dementia and their accompanying family members are informed about the facilities that can help caring for the patients. Helpful leaflets, addresses, phone number and web sites are provided. Next, most patients are referred back to their General Practitioner. About a quarter of Alzheimer patients is prescribed anti-Alzheimer medication, and in such cases patient and family caregiver visit the memory clinic regularly for medication control visits. Family meetings are organized rarely and never in a structured way and with follow-ups. Moreover they tend to focus on providing clinical information and not on increasing family support.

In contrast with memory clinics, most mental health care institutions do offer support for caregivers of dementia patients. The kind of support differs per institution. This varies from support groups for caregivers to case management for active support and organization of needed care. Just like memory clinics, mental health care institutions rarely organize family meetings and never in a structured way and with follow-ups.

### Measurements

Table 2 provides an overview of all effect and economic measurements. Before baseline, caregivers will be screened on the presence of a depressive or anxiety disorder. This measurement is repeated every 3 months (at T1, T2, T3 and T4). At baseline (T0) and after 6 (T2), 12 (T4) months participants are interviewed by trained interviewers. The interviewers leave cost diaries for caregiver and patient to register medical consumption. When patients are unable to answer the questions their informal caregiver is allowed to answer or to provide assistance.

**Table 2:** Measurement Scheme

**Variable**	**Instrument**	**T-1**	**T0**	**T1**	**T2**	**T3**	**T4**
**Effect evaluation: primary outcomes**							
Depressive & anxiety disorders	MINI	X		X	X	X	X
Severity depression	CES-D, GDS-5		X		X		X
Severity anxiety	HADS anxiety subscale		X		X		X
**Effect evaluation: secondary outcomes**							
Quality of life caregiver	SF-12		X		X		X
Caregiver burden	SSCQ, CRA, SRB		X		X		X
Social support in caregiver	Questions based on assessment battery study		X		X		X
Days until institutionalisation patient	according to carer	Continuous registration					
**Economic evaluation**							
Direct and indirect costs carer + patient	Cost diaries		X		X		X

T0 = baseline

T1 = 3 months after baseline

T2 = 6 months after baseline

T3 = 9 months after baseline

T4 = 12 months after baseline

#### Effect evaluation

*Primary outcomes *are:

1. Incidence of major depression and anxiety disorders (generalised anxiety, panic, social phobia, agoraphobia, obsessive compulsive disorder, hypochondria) as defined according to DSM-IV criteria [23] and assessed with the Mini International Neuropsychiatric Interview (MINI) [24]. The MINI is used as 3 month prevalence measure.

2. The severity of anxiety and depression symptoms in carers is measured by validated self report instruments. For depression we use the Centre for Epidemiologic Studies Depression Scale (CES-D) [25]. For anxiety, the anxiety scale from the Hospital Anxiety and Depression Scales (HADS) [26,27] is used.

*Secondary outcomes *are:

3. Caregiver burden with the Short Sense of Competence Questionnaire (SSCQ) [28], the Caregiver Reaction Assessment (CRA) [29] and a single self-rated burden question (SRB) [30].

4. Caregiver's quality of life with the Short Form 12 item version[31,32].

5. Social support caregiver (see below)

6. Days until institutionalization of the patient

One of the main goals of the intervention is to mobilize support of naturally existing family networks. Therefore social support of caregiver is measured with a Social Support Assessment based on the Caregiver Assessment Battery used in the studies of Mittelman [20]. In this assessment the caregiver is asked how satisfied he/she is with his or her social network in general, if he/she is satisfied with the received amount of emotional and practical support and if this received support is useful. In addition, loneliness is measured with the loneliness scale [33].

##### Potential effect modifiers

Besides the primary and secondary outcomes, we evaluate several variables which we assume as potential effect modifiers. The following variables on the level of the caregiver are assessed: socio-demographic characteristics, characteristics of the caregiving situation (family relation between patient and caregiver, duration and hours of caregiving, type of caregiving), presence of chronic diseases.

On patient level we assess socio-demographic characteristics, cognitive functioning with the MMSE [34], type of dementia, depressive and behavioral symptoms with the Neuropsychiatric Inventory (NPI)[35], patient's quality of life as measured with the SF-12, (Instrumental) Activities of daily living [36,37], medical co-morbidity and treatment and formal care support.

##### Additional measures

In order to take note of the kind of problems dementia caregivers have to deal with, we used the Psychological Outcomes Profiles (PSYCHLOPS). This is a practical simple tool that focuses on problems respondents experience. With the PSYCHLOPS we evaluate if problems of caregivers disappear during the intervention or are substituted by other problems.

Besides the CES-D to measure depressive symptoms in caregivers, we added the Geriatric Depression Scale (GDS-5) [38] to be able to integrate data with the intervention study of Mittelman.

#### Process evaluation

A process evaluation will be included to help interpret the outcome results. Furthermore, this process evaluation has the following aims:

- to document how the intervention is implemented and to what extent the counselors adhered to the protocol. Because of the flexible character of the intervention it is important to record what really happened during the family meetings.

- to examine the views of participating caregivers and counselors on the intervention.

- to distinguish between key components of the intervention. This can make clear through which mechanisms the intervention may work and which components of the intervention influence the study outcomes most.

We use several methods to collect process data:

- counselors complete a standard form after every session. This form asks for the session characteristics (duration, number of persons attending the meeting, attendance of the patient) and content of the session (which problems have been discussed, goals for the next session). After the first and last session the counselor also provides some background information about the family (level of knowledge, family conflicts) and successfulness of the intervention for the family according to the counselor.

- we check if every family in the intervention group received the planned number of four sessions and if not, the primary caregiver or counselor is asked for the reason.

- after the last session caregivers as well as counselors are interviewed to explore their opinions on the intervention.

- all sessions are recorded to be able to evaluate other aspects.

#### Economic evaluation

Six monthly cost diaries collect cost data from a societal perspective. The evaluation is a combination of a cost-effectiveness analysis on caregiver's depression and anxiety and cost-utility analysis on caregivers and patients separately. Health-related utilities and in consequence quality-adjusted life-years (QALYs) were measured using the SF-6D, which is calculated using a sub-set of questions from the SF-12[39]. If patients are not capable to answer the questions their caregiver will rate the patients' health on the SF-12.

The following costs of both caregiver and patient will be considered: 1) direct healthcare costs, such as costs of the family meetings, consultations of the general practitioner, nursing home physician, medical specialist, hospitalizations, and medical department of the nursing home, and use of medication and medical aids; 2) direct non-healthcare costs (travel time and costs, informal care time); 3) indirect costs (costs of lost labour days of working caregivers). If available, Dutch guideline prices are used to value resource use [40]. Otherwise, tariffs are used. A cost price for the family meetings will be calculated using a bottom-up approach. Medication costs are valued using prices of the Royal Dutch Society for Pharmacy (Z-index 2004). Lost productivity costs are calculated according to the friction cost approach (friction period 154 days) using the mean age and sex specific income of the Dutch population [41,42].

### Sample size calculations

Sample size calculations are based on the expected effects of the intervention on the main outcome measures, incidence of affective disorders and increasing time to incident problems. Incidence estimates are derived from the literature and other studies performed by our group (Prevention of anxiety and depression in late life). Effect estimates are based on the primary studies of Mittelman [17,19]. We estimate the yearly incidence of affective disorders among caregiver at risk conservatively at 30% [7]. We aim to reduce the incidence to 10% of new cases a year. With an alpha of 0.05, power of 80%, 73 persons per group are then needed. Assuming 15% loss to follow up we need to recruit 2 × 73 × 100/85 = 172 pairs of dementia patients and family caregivers. This number will be sufficient as well to measure a moderately strong reduction (Cohen's D > 0.5) in number of depressive or anxiety symptoms.

### Blinding

Baseline and follow up data are gathered by independent and blinded interviewers. Participants are not blinded.

### Analysis

#### Effect evaluation

Data will be primarily analyzed according to the Intention to treat principle, i.e. including all participants with valid data, regardless of whether they did or did not receive the intervention. Subsequently, the results of the intention to treat analysis will be compared with the results of the 'on treatment' analysis, to assess whether protocol violations have caused bias. Participants with documented deviations from the protocol (i.e. participants who did not receive the entire intervention or participants in either the intervention or the control group with incomplete follow-up data) will be excluded from the on treatment analysis. Comparability between the intervention and usual care groups will be assessed at baseline to check differences.

Intervention effects will be analyzed with survival analysis (Cox Proportional Models) for the time to incident affective disorder and repeated measurement analyses for number of symptoms. Both techniques can adjust for possible baseline imbalance, and potential modifiers (e.g. dementia type and severity, gender caregiver, relation with patient, patient centre/counselor, family type (e.g. conflictions versus harmonious, close versus distant). Dropout and loss to follow up will be described.

#### Economic evaluation

To compare costs between the two groups, confidence intervals for the differences in mean costs are calculated using bias-corrected and accelerated bootstrapping with 2000 replications [43]. For the cost-effectiveness analysis the difference in total costs between the intervention and usual care group are compared with the difference in psychological problems and for the cost-utility analysis with the difference in the number of QALYs gained after 24 months. Uncertainty around the cost-effectiveness and cost-utility ratios will be calculated using bias-corrected percentile bootstrapping method with 5000 replications [44]. The bootstrapped cost-effect pairs will be plotted on cost-effectiveness planes and will be used to calculate cost-effectiveness acceptability curves.

## Discussion

In this paper we described the study protocol of an innovative RCT that evaluates (cost)-effectiveness of family meetings on indicated prevention of anxiety and depressive disorders and symptom levels of primary informal caregivers of dementia patients who live at home.

A multi-component intervention including family meetings led to sustained benefits in reducing depressive symptoms in caregivers as well as in postponement of nursing home placement of Alzheimer's disease patients {Mittelman, 2004 135/id}{Mittelman, 2006 133/id}.

However, the effectiveness of using only the family counseling component of this intervention has not been tested. Furthermore, effects of family meetings were not replicated in other countries yet. By evaluating the effectiveness of only structured family meetings organized in the Netherlands, this study will contribute to the existing literature about the value of psychosocial interventions for dementia caregivers. In addition, this study involves some other innovative elements. The New York University Spouse-Caregiver Intervention {Mittelman, 2003 70/id} was done with caregivers of Alzheimer type dementia patients only and measured effects on depression but not on anxiety. In our study we also include non-Alzheimer type dementias and measure both depression and anxiety.

Besides these innovating elements which are a particular strength of this study, we can also think of some limitations or potential threats.

Selection of participants might be a possible limitation of the study, because we only include patients living together with a family caregiver and another supporting person in their social network. This has consequences for the generalizability of the results to other kind of situations, for example patients living alone and having a child who takes primary responsibility. It is also possible that counselors instinctively exclude some kind of families, for example because they think families are not suitable or expect that they are not willing to participate.

A possible threat to achieve sufficient contrast exists when (unstructured) family counseling will become part of the usual care in some participating centers. So far, only in some centers disclosure meetings are organized with family members. In the recruitment phase, we will pay specific attention to this to be sure that participating centers will not yet organize family meetings in a frequent and structured way.

Because of our widely formulated inclusion criteria, and limited exclusion criteria we expect heterogeneity in study subjects. For example, it is likely that caregiving intensity and ditto stress is differently for caregivers of patients in the beginning of the dementia than for caregivers of patients who are already in an advanced stadium. It is also possible that the different types of dementia influences the caregiving situation. This may substantially modify the outcomes. Therefore it is important to include enough study subjects and carry out an active recruitment of centers for participation in the study.

The Alzheimer Center of the VUmc has currently begun to pilot family meetings. The first experiences with families of recently diagnosed patients were very encouraging. When effective, family meetings can be an important addition to the current care services in the Netherlands. The results of this study will be available in the beginning of 2011.

## Competing interests

The author(s) declare that they have no competing interests.

## Authors' contributions

KJ conducts the research and wrote this article. HH is principal investigator, writer of the study protocol and supervises the project. PS is head of The Alzheimer Center of the Vumc and supervises the project. FG is a dementia nurse at The Alzheimer Center of the VUmc and involved with training and supervision of the counselors. JB and BB perform the economic evaluation. MVD advised on the content and design of the study. MM developed the intervention that was the inspiration for this study. HM is supervisor and writer of the study protocol. All authors provided comments on the drafts and have read and approved the final version of the manuscript.

## Pre-publication history

The pre-publication history for this paper can be accessed here:


